# Renal outcomes of rivaroxaban compared with warfarin in Asian patients with nonvalvular atrial fibrillation: A nationwide population-based cohort study

**DOI:** 10.3389/fcvm.2023.1040834

**Published:** 2023-02-23

**Authors:** So-Ryoung Lee, Eue-Keun Choi, Sang-Hyun Park, Kyung-Do Han, Seil Oh, Khaled Abdelgawwad, Gregory Y. H. Lip

**Affiliations:** ^1^Department of Internal Medicine, Seoul National University Hospital, Seoul, Republic of Korea; ^2^Department of Internal Medicine, Seoul National University College of Medicine, Seoul, Republic of Korea; ^3^Department of Medical Statistics, College of Medicine, Catholic University of Korea, Seoul, Republic of Korea; ^4^Statistics and Actuarial Science, Soongsil University, Seoul, Republic of Korea; ^5^Bayer AG, Berlin, Germany; ^6^Liverpool Centre for Cardiovascular Science, Liverpool Chest and Heart Hospital, University of Liverpool, Liverpool, United Kingdom; ^7^Department of Clinical Medicine, Aalborg University, Aalborg, Denmark

**Keywords:** warfarin, kidney failure, atrial fibrillation, anticoagulation, rivaroxaban

## Abstract

**Background:**

Further studies are needed to expand the evidence for the association of rivaroxaban with a lower risk of adverse renal outcomes in patients with atrial fibrillation (AF) as compared with warfarin, especially in Asians.

**Objectives:**

To determine whether there are differences in adverse renal outcomes between rivaroxaban and warfarin-treated AF patients.

**Methods:**

Using the Korean nationwide claims database partly linked to laboratory results, patients with AF who initiated warfarin or rivaroxaban from 1 January 2014 to 31 December 2017 were identified. Inverse probability of treatment weighting (IPTW) was used to balance the baseline characteristics of the two groups. The primary outcome (kidney failure) was defined as the need for maintenance dialysis or having kidney transplantation. For the exploratory analysis in a subset of patients with baseline and follow-up laboratory results, the composite of renal outcomes, including estimated glomerular filtration rate (eGFR) lower than 15 ml/min/1.73 m^2^ at follow-up measurement, starting dialysis, or having kidney transplantation, ≥ 30% decline in eGFR, doubling of serum creatinine level, and acute kidney injury (AKI) were evaluated. The two groups were compared using Cox proportional hazards regression in the weighted population.

**Results:**

We identified 30,933 warfarin users and 17,013 rivaroxaban users (51% of low dose rivaroxaban). After IPTW, the mean age was 70 years, and the mean CHA_2_DS_2_-VASc score was 3.9 in both groups. During a median follow-up of 0.93 (interquartile ranges 0.23–2.10) years, weighted incidence rates of kidney failure for warfarin and rivaroxaban were 0.83 and 0.32 per 100 person-years, respectively. Compared with the warfarin group, the rivaroxaban group was associated with a lower risk of kidney failure (hazard ratio [HR] 0.389, 95% confidence interval [CI] 0.300–0.499, *p* < 0.001). In patients with preexisting chronic kidney disease or eGFR ≤ 60 ml/min/1.73 m^2^, rivaroxaban was more beneficial than warfarin in reducing the risk of kidney failure. For the composite of five renal outcomes in the exploratory analysis, the rivaroxaban group showed a lower risk than warfarin (HR 0.798, 95% CI 0.713–0.892, *p* < 0.001).

**Conclusion:**

Rivaroxaban was associated with lower risks of renal adverse outcomes than warfarin in Korean patients with AF.

## Introduction

Atrial fibrillation (AF) has been related to an increased risk of chronic kidney disease (CKD) later in life (1). For several decades, warfarin was the only oral anticoagulation (OAC) therapy in preventing thromboembolic events in AF patients. Warfarin-related nephropathy, including the rapid development of renal function decline in CKD patients and the prevalence of acute kidney injury (AKI), has been described among warfarin-treated patients (2, 3).

Since the introduction of non-vitamin K antagonist oral anticoagulants (NOACs), there has been some evidence that NOACs might be associated with improved renal function preservation compared with warfarin (4, 5). According to a post-hoc analysis of the Randomized Evaluation of Long-Term Anticoagulation Therapy (RE-LY) trial, dabigatran was linked to a reduced risk of creatinine clearance reduction compared with warfarin (4). Several observational studies found that NOACs had similar results to warfarin, but there was variance in the outcomes across NOACs (5–9). Firstly, rivaroxaban and dabigatran consistently outperformed warfarin regarding kidney preservation (5–9). On the other hand, apixaban did not produce consistent findings with statistical significance (5), and there was no information on edoxaban. Secondly, the relationship between NOAC and the likelihood of unfavorable renal outcomes varied depending on the patients’ baseline kidney function (10). Finally, between non-Asians and Asians, the protective effect of NOACs versus warfarin on renal outcomes was slightly different (9).

Renal function deterioration is widespread in AF patients treated with OAC (5). As decreased renal function is associated with an increased risk of stroke and bleeding, it is critical to maintain renal function in patients treated with OAC (11, 12). Further studies are needed to examine whether NOACs bring consistent results for preventing progressive renal function decline, especially in Asians who had poor treatment quality of warfarin therapy (13).

This study aims to determine whether there are differences in adverse renal outcomes between rivaroxaban and warfarin-treated AF patients utilizing a nationwide population-based study in South Korea.

## Materials and methods

### Data source

This retrospective observational nationwide population-based cohort study was conducted using administrative claims data from the Korean National Health Insurance Service (NHIS) and the linked health check-up database of the National Health Insurance Corporation (NHIC) (14, 15). The Korean NHIS provides comprehensive medical care coverage for the entire Korean population (approximately 50 million people). The analysis was based on a randomly selected 50% sample cohort from the Korean NHIS. Supplementary methods provide additional information about the data source. All data have been provided publicly available through the National Health Insurance Data Sharing Service (accessed at: http://nhiss.nhis.or.kr/bd/ab/bada000eng.do). After permission to use the data was obtained, the analysis was performed at the Korean NHIS Big Data Center, Seoul, Republic of Korea.

### Study population and study design

**Figure 1:**
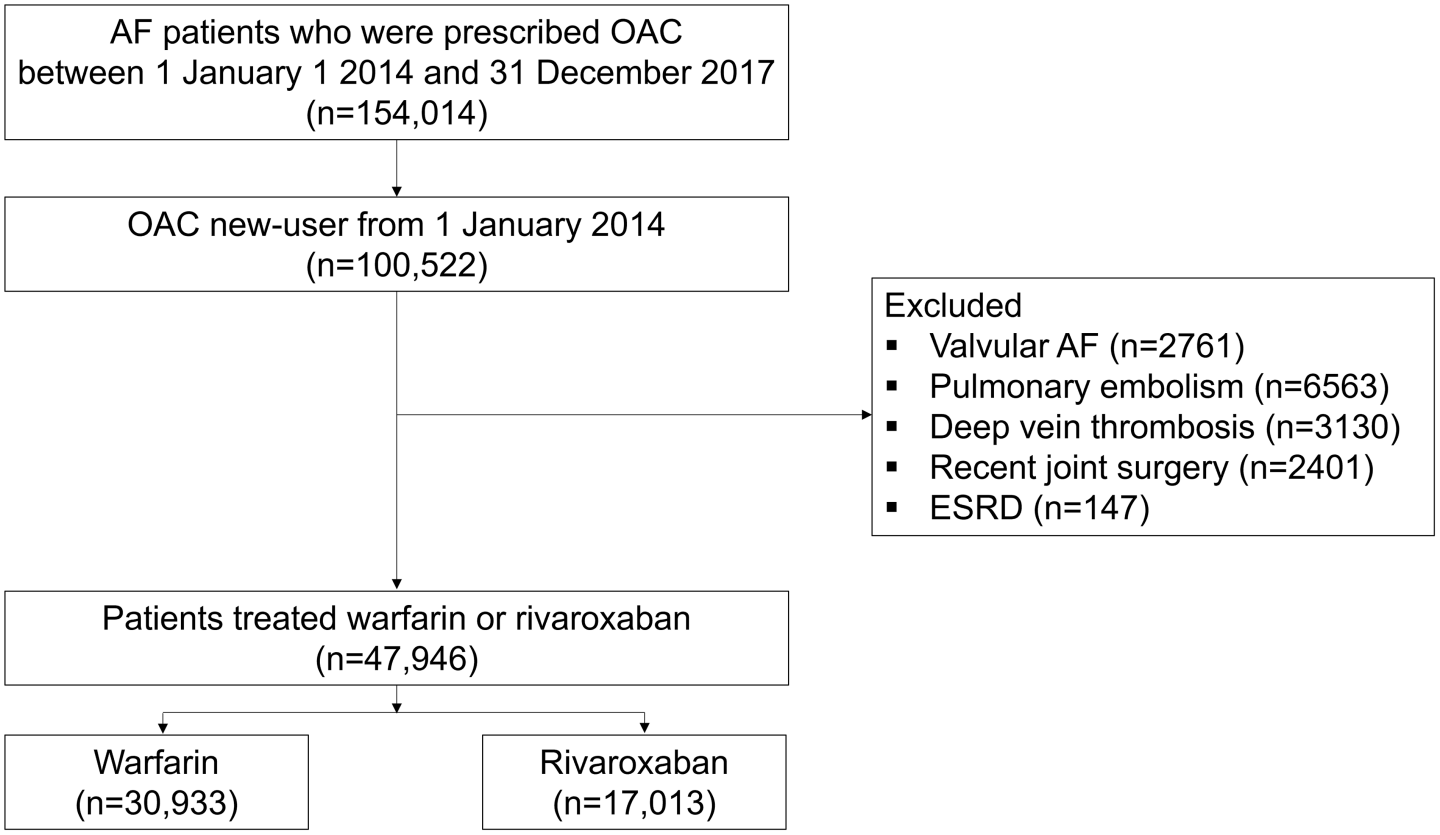
Study enrollment flow. AF, atrial fibrillation; ESRD, end-stage renal disease; OAC, oral anticoagulant.

The study period was from 1 January 2013 to 31 December 2018. The study’s enrollment period ran from 1 January 2014 to 31 December 2017, to allow for at least a 12-month follow-up period. Study enrollment flow is presented in Figure 1. Firstly, we identified adult AF patients prescribed OAC during the enrollment period. AF was defined as at least one hospitalization or outpatient visit with relevant diagnostic codes (I48.0–I48.4, I48.9). To compare the renal outcome between two treatment groups (rivaroxaban versus warfarin), we included patients who were OAC new users (who had no record of OAC use in the prior 12 months) and were newly initiated on rivaroxaban or warfarin. Patients with valvular AF, alternative indications of OAC including pulmonary embolism, deep vein thrombosis, recent joint surgery, and end-stage renal disease (ESRD) were excluded.

The primary analysis included all eligible patients. Additionally, we designed the exploratory analysis to assess renal outcomes estimated by laboratory data, including a subset of patients who received at least two health examinations during the study period. These patients had baseline and follow-up eGFR measurements. As a baseline eGFR, we collected the results of the health examination performed within 2-year from the index date. Among patients with a baseline eGFR value, we included patients with at least one follow-up health examination data during follow-up.

### Covariates

Age, sex, co-morbidities including hypertension, diabetes, dyslipidemia, heart failure, prior stroke, prior myocardial infarction, peripheral artery disease, chronic kidney disease, chronic obstructive pulmonary disease (COPD), and cancer, CHA_2_DS_2_-VASc score, Charlson Comorbidity Index (CCI), and concomitant use of antiplatelet agents were evaluated as covariates. The operational definitions of co-morbidities were based on diagnostic codes, drug dispensing records, and inpatient/outpatient hospital visits within 3 years prior to the index date. Complete definitions of each covariate are presented in Supplementary Tables S1 and S2 (5, 15, 16).

Among the total study population, 67.4% of patients had the data from the baseline national health examination, and 23.4% had the data from both baseline and at least one follow-up national health examination. From the health examination data, body weight, body mass index (kg/m^2^), serum creatinine (mg/dL) and eGFR (mL/min/1.73 m^2^) were collected. eGFR was calculated by a creatinine-based equation used from Modification of Diet in Renal Disease. In addition, smoking status (never smoker, ex-smoker, or current smoker), alcohol consumption (heavy drinker, ≥ 30 g/day), and physical activity were also evaluated from the self-reported questionnaires of health examination. Regular exercise was defined as performing moderate-intensity exercise ≥ 5 times per week or vigorous-intensity exercise ≥ 3 times per week (17).

### Study outcomes and follow-up

The index date was defined as the time when rivaroxaban or warfarin was newly initiated. To evaluate the comparative risk of renal outcome between the two groups, the primary outcome was incident kidney failure, defined as the need for maintenance dialysis or having kidney transplantation (Supplementary Table S3) (5, 18). Secondary outcomes were incident ischemic stroke, intracranial hemorrhage, major gastrointestinal bleeding, major bleeding, and all-cause death (Supplementary Table S3) (16). To assess the outcomes, patients were followed up until 31 December 2018. Patients were censored at the occurrence of each outcome, the end of the study period (31 December 2018), or death, whichever came first. In addition, the main analysis followed the on-treatment approach; therefore, patients were also censored at the discontinuation of index treatment for more than 30 days. The date of discontinuation was defined as the end of exposure, and patients were censored.

For the exploratory analysis, five renal outcomes were assessed; [1] eGFR lower than 15 ml/min/1.73 m^2^ at follow-up measurement, [2] starting dialysis or having kidney transplantation, [3] ≥ 30% decline in eGFR, [4] doubling of serum creatinine level, and [5] AKI (Supplementary Table S3) (5). The 30% decline in eGFR and doubling of serum creatinine defined as changes from baseline (using measurement closest to index date) at any time point during follow-up (5). Because [1, 3, 4] relied entirely on laboratory data, when examining these three outcomes, patients were censored at their last laboratory measurement. AKI was defined as an emergency department visit or hospitalization with a diagnostic code of AKI (N17 ×) (5, 9). The composite of five renal outcomes was also evaluated.

### Statistical analysis

Patients were described at treatment initiation in terms of demographic and clinical variables. Continuous variables are presented as means and standard deviations or medians and interquartile ranges (IQR). The numbers and proportions of patients in each category are presented for categorical variables. Person-years of follow-up were calculated from the index date to the outcome event of interest, discontinuation of the index treatment, death, or the end of the study period, whichever comes first. Incidence rates were calculated as the number of events over the observed person-time and presented as per 100 person-years.

We used the propensity score (PS) methods to compare the rivaroxaban and warfarin groups (19). We utilized stabilized inverse probability of treatment weighting (IPTW) approach based on the PS to adjust for potential confounding resulting from imbalances in baseline patient characteristics. The objective of IPTW is to create a weighted sample for which the distribution of either the confounding variables or the prognostically important covariates is approximately the same between comparison groups (20). PS is the patient’s probability of receiving a treatment under investigation (rivaroxaban) given a set of known patients’ baseline characteristics. PS was calculated using multiple logistic regression on all the available covariates, including demographics, co-morbidities, CHA_2_DS_2_-VASc score, Charlson Comorbidity Index, and concomitant medication. For the exploratory analysis, health examination variables such as body weight, body mass index (BMI), eGFR, smoking, alcohol consumption, and physical activity were additionally included for PS calculation. Detailed methods of IPTW are described in Supplementary methods. After IPTW, we assessed the balance of the two treatment groups by using absolute standardized differences (ASDs). The PSs and stabilized weights distributions were inspected for initial and synthetic samples. An ASD of 0.1 or less was considered as a negligible difference between the two groups. The weighted event numbers and incidence rates were calculated. We compared treatments using weighted Cox proportional hazards regression with IPTW. Results of Cox analyses are reported as hazard ratios (HRs) with 95% confidence intervals (CIs). Each Cox regression was checked to see if the model assumptions were fulfilled. For the exploratory analysis set, weighted cumulative incidences of the composite of five renal outcomes were estimated by the Kaplan–Meier method and log-rank test.

All statistical analyses were performed using SAS 9.3 (SAS Institute Inc., Cary, NC, United States).

### Subgroup analyses

In the main analysis set, for the primary outcome, subgroup analyses were performed for age strata (< 65, 65–74, and ≥ 75 years), sex, hypertension, diabetes, heart failure, CKD (defined by diagnostic codes), CHA_2_DS_2_-VASc score (< 3, and ≥ 3) and Charlson Comorbidity Index (< 3, and ≥ 3). Among patients with baseline eGFR measurements, subgroup analyses were performed for eGFR ranges (> 60 and ≤ 60 ml/min/1.73 m^2^). Subgroup analyses were performed using a multivariable Cox proportional hazards regression model. The variables used in the multivariable Cox analysis were identical to those used in the PS calculation for the main analysis set. Tests for interaction were conducted to evaluate statistically significant (*p* < 0.1) subgroup differences in treatment.

### Sensitivity analyses

To provide complementary analyses, we performed sensitivity analyses for the primary outcome as follows: [1] IPTW following the intention-to-treat (ITT) approach, which was not censoring patients at discontinuation or switching the index treatment), [2] multivariable Cox proportional hazards regression models in the study population before IPTW following the on-treatment approach, [3] multivariable Cox analysis following the ITT approach, [4] 5% trimmed IPTW following the on-treatment approach, [5] 5% trimmed IPTW following the ITT approach, [6] a sensitivity analysis among patients with a 6-month or longer follow-up period to evaluate whether the main results are consistent in those who had neither drug discontinuation nor any renal outcome during the first 6 months, [7] a sensitivity analysis restricting the follow-up within 12 months, and [8] an analysis in the subset of patients with baseline eGFR measurements. The sensitivity analyses of [2, 3, 6, 7] were performed using a multivariable Cox proportional hazards regression model, and the variables used in the multivariable Cox analysis were identical to those used in the PS calculation for the main analysis set. For [8], baseline eGFR values were additionally adjusted. In addition, although we included the CHA_2_DS_2_-VASc score and CCI in the final multivariable Cox analysis, there is a possibility of model overfitting. Therefore, we conducted a sensitivity analysis excluding the CHA_2_DS_2_-VASc score, CCI, or both of these in the final model. Also, we performed a competing risk analysis with the Fine-Gray methods as a sensitivity analysis (21).

## Results

### Baseline characteristics

This study comprised a total of 47,946 individuals (mean age 70.1 ± 11.7 years, mean CHA_2_DS_2_-VASc score 3.9 ± 1.9), with 30,933 patients taking warfarin and 17,013 taking rivaroxaban. Supplementary Table S2 shows the baseline characteristics of the total, warfarin, and rivaroxaban groups. Before PS matching, the rivaroxaban group was older, more likely to be women, and had a higher mean CHA_2_DS_2_-VASc score than the warfarin group. Co-morbidities such as hypertension, diabetes, dyslipidemia, heart failure, peripheral artery disease, and cancer were more common in the rivaroxaban group. In contrast, prior stroke, prior myocardial infarction, chronic kidney disease, and chronic obstructive pulmonary disease were more prevalent in the warfarin group. Antiplatelet co-use was more common in the warfarin group than in the rivaroxaban group. In the rivaroxaban group, standard dose rivaroxaban (20 mg once daily) was prescribed to 49% of patients, whereas low-dose rivaroxaban (15 mg once daily) was prescribed to 51%.

### Primary and secondary outcomes

In the main analysis set, a median follow-up duration was 0.93 (IQR 0.23–2.10) years. Rivaroxaban group showed longer median follow-up duration than warfarin group (1.27 [IQR 0.27–2.35] vs. 0.75 [0.21–1.85], *p* < 0.001). Supplementary Table S4 shows crude event numbers, incidence rates, and unadjusted HRs for primary and secondary outcomes. In Table 1, all baseline variables were well-balanced in the two groups after PS weighting, and all ASDs for the two groups were less than 0.1 (Table 1). PS distribution after weighting is presented in Supplementary Figure S1.

**Table 1:** Baseline characteristics of warfarin and rivaroxaban groups before and after inverse probability of treatment weighting (IPTW).

	Before IPTW			After IPTW		
	Warfarin	Rivaroxaban	ASD	Warfarin	Rivaroxaban	ASD
n	30,933	17,013		30,946	17,006	
Age, years	69.0 ± 12.3	72.1 ± 10.1	0.277	70.2 ± 11.9	70.4 ± 11.2	0.015
< 65 years	9,944 (32.2)	3,468 (20.4)		87,223 (28.2)	4,523 (26.6)	
65 to < 75 years	9,412 (30.4)	5,974 (35.1)		9,700 (31.3)	5,701 (33.5)	
≥ 75 years	11,577 (37.4)	7,571 (44.5)		12,524 (40.5)	6,782 (39.9)	
Sex, male	18,260 (59.0)	9,605 (56.5)	0.052	17,985 (58.1)	9,909 (58.4)	0.003
CHA_2_DS_2_-VASc	3.8 ± 2.0	4.1 ± 1.7	0.125	3.9 ± 1.9	3.9 ± 1.9	0.010
CHA_2_DS_2_-VASc ≥ 3	22,494 (72.7)	13,779 (81.0)	0.197	23,446 (75.8)	12,949 (76.2)	0.013
Charlson comorbidity index	4.0 ± 2.5	4.0 ± 2.4	0.006	4.0 ± 2.5	4.0 ± 2.5	0.015
Charlson comorbidity index ≥ 3	21,444 (69.3)	11,978 (70.4)	0.023	21,668 (70.0)	11,900 (70.0)	0.009
Hypertension	25,023 (80.9)	14,582 (85.7)	0.129	25,572 (82.6)	14,050 (82.6)	<0.001
Diabetes	8,067 (26.1)	4,617 (27.1)	0.023	8,213 (26.5)	4,558 (26.8)	0.005
Dyslipidemia	16,290 (52.7)	9,357 (55.0)	0.046	16,563 (53.5)	9,150 (53.8)	0.005
Heart failure	12,550 (40.6)	7,592 (44.6)	0.082	12,988 (42.0)	7,104 (41.8)	0.003
Prior stroke	9,511 (30.8)	4,315 (25.4)	0.120	8,964 (29.0)	5,017 (29.5)	0.011
Prior myocardial infarction	2026 (6.6)	1,004 (5.9)	0.026	1955 (6.3)	1,079 (6.3)	0.001
Peripheral artery disease	6,948 (22.5)	4,355 (25.6)	0.073	7,301 (23.6)	4,025 (23.7)	0.001
Chronic kidney disease	1899 (6.1)	724 (4.3)	0.084	1,699 (5.5)	976 (5.7)	0.010
COPD	2,975 (9.6)	1,372 (8.1)	0.054	2,814 (9.1)	1,578 (9.3)	0.006
Cancer	2003 (6.5)	1,368 (8.0)	0.060	2,177 (7.0)	1,219 (7.2)	0.005
Antiplatelet use						
None	18,790 (60.7)	12,679 (74.5)	0.235	20,305 (65.6)	11,114 (65.4)	<0.001
Aspirin only	6,562 (21.2)	2,137 (12.6)		5,611 (18.1)	3,090 (18.2)	
P2Y_12_ only	1889 (6.1)	902 (5.3)		1800 (5.8)	1,002 (5.9)	
Both	3,701 (12.0)	1,295 (7.6)		3,230 (10.4)	1800 (10.6)	
Rivaroxaban dose						
20 mg once daily	N/A	8,354 (49.1)		N/A	8,022 (47.2)	
15 mg once daily	N/A	8,659 (50.9)		N/A	8,984 (52.8)	

Continuous variables are shown as mean and standard deviation. Categorical variables are presented as numbers (percentages).

ASD, absolute standardized difference; CHA_2_DS_2_-VASc, congestive heart failure, hypertension, age ≥ 75 (doubled), diabetes mellitus, prior stroke or transient ischemic attack (doubled), vascular disease, age 65–74, female; COPD, chronic obstructive pulmonary disease; IPTW, inverse probability of treatment weighting; N/A, not available.

**Figure 2:**
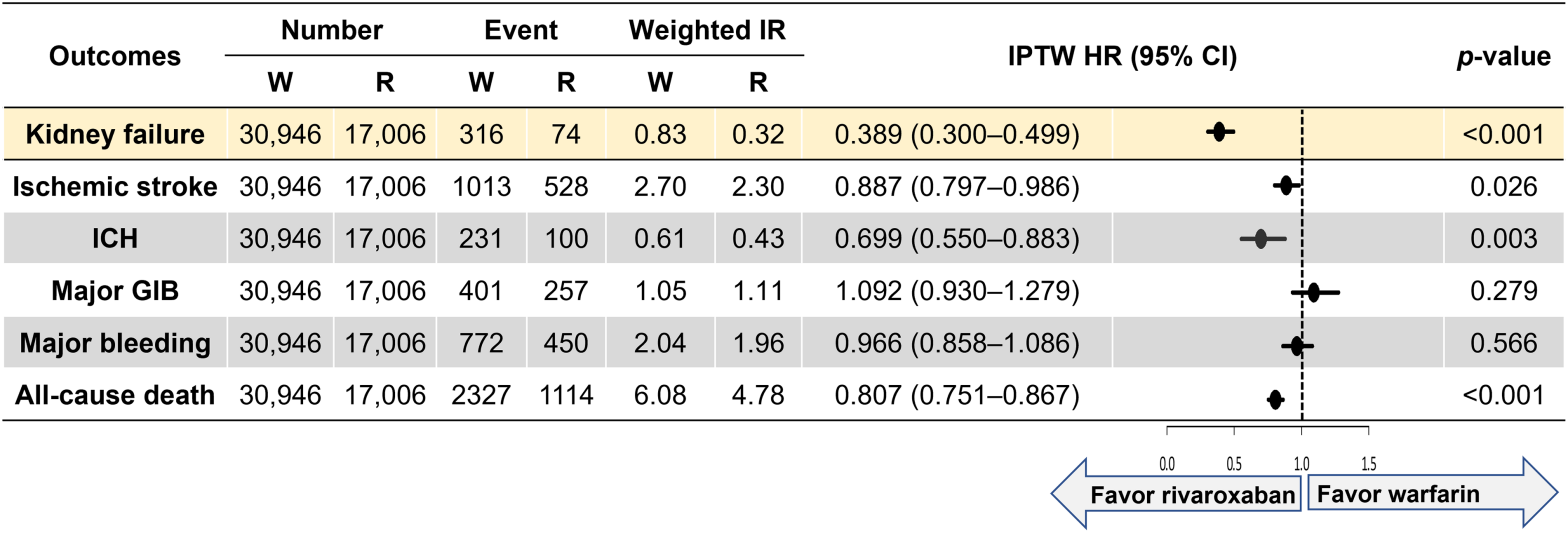
Weighted event numbers, incidence rates, and hazard ratios for the primary and secondary outcomes between warfarin and rivaroxaban groups. Incidence rate, per 100 person-years. CI, confidence interval; GIB, gastrointestinal bleeding; ICH, intracranial hemorrhage; IPTW, inverse probability of treatment weighting; IR, incidence rate; R, rivaroxaban; W, warfarin.

Figure 2 shows weighted incidence rates and weighted HRs for primary and secondary outcomes. Compared with the warfarin group, the rivaroxaban group was associated with a lower risk of kidney failure (HR 0.398, 95% CI 0.300–0.499). For the secondary outcomes, the rivaroxaban group was associated with lower risks of ischemic stroke (HR 0.887, 95% CI 0.797–0.986), intracranial hemorrhage (HR 0.699, 95% CI 0.550–0.883), and all-cause death (HR 0.807, 95% CI 0.751–0.867) than the warfarin group. The two groups had comparable outcomes for major gastrointestinal bleeding (HR 1.092, 95% CI 0.930–1.279) and major bleeding (HR 0.966, 95% CI 0.858–1.086).

### Sensitivity analyses

For the primary outcome we performed various sensitivity analyses that demonstrated results consistent with the main analysis. Rivaroxaban was associated with significant reductions in the risk for kidney failure in all analyses (Supplementary Results, Supplementary Figure S2, and Supplementary Table S5). The results were consistent with the primary findings when we conducted a competing risk analysis that was adjusted for the competing risk of death rather than a censoring event (HR 0.447, 95% 0.344–0.582, *p* < 0.001).

### Subgroup analyses

**Figure 3:**
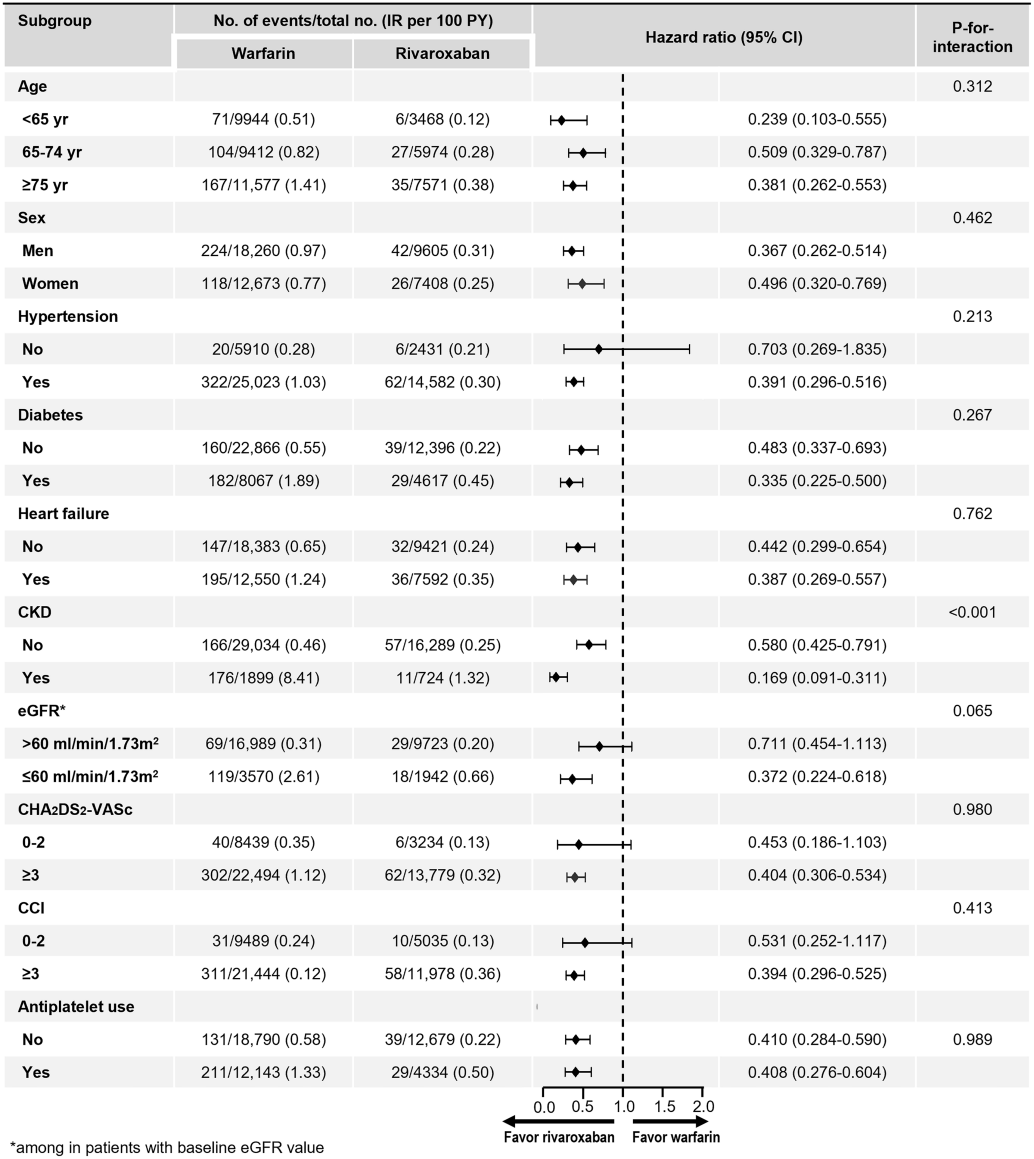
Subgroup analyses. CCI, Charlson comorbidity index; CI, confidence interval; CKD, chronic kidney disease; eGFR, estimated glomerular filtration rate; IR, incidence rate.

The benefit of rivaroxaban compared with warfarin on the risk of kidney failure was consistently observed across almost all of the examined subgroups (Figure 3). However, wide CI was observed in patients without hypertension due to the small number of patients and low event rates. There were no significant interactions between treatment and all subgroups, except in the subgroup stratified by CKD and eGFR. Rivaroxaban was associated with a greater reduction in the risk of kidney failure in patients with underlying CKD, as defined by diagnostic codes, compared with those without (value of *p* for interaction < 0.001). There was also a strong trend towards a reduction in the risk of kidney failure in patients with CKD defined as eGFR less than 60 ml/min/1.73 m^2^, compared with those with an eGFR greater than 60 ml/min/1.73 m^2^.

### Exploratory analysis in patients with baseline and follow-up eGFR measurements

Among the total study population, 11,210 (23.4%) patients were included in the exploratory analysis. Baseline characteristics of the total population, warfarin, and rivaroxaban group are presented in Supplementary Table S6. After IPTW, the two groups were well-balanced in all variables (all ASDs < 0.1). Mean baseline eGFR was 81.6 ml/min/1.73 m^2^ in the two groups (ASD < 0.001). The duration from baseline eGFR to index date and baseline eGFR to follow-up eGFR of the two groups did not show statistically significant differences.

**Figure 4:**
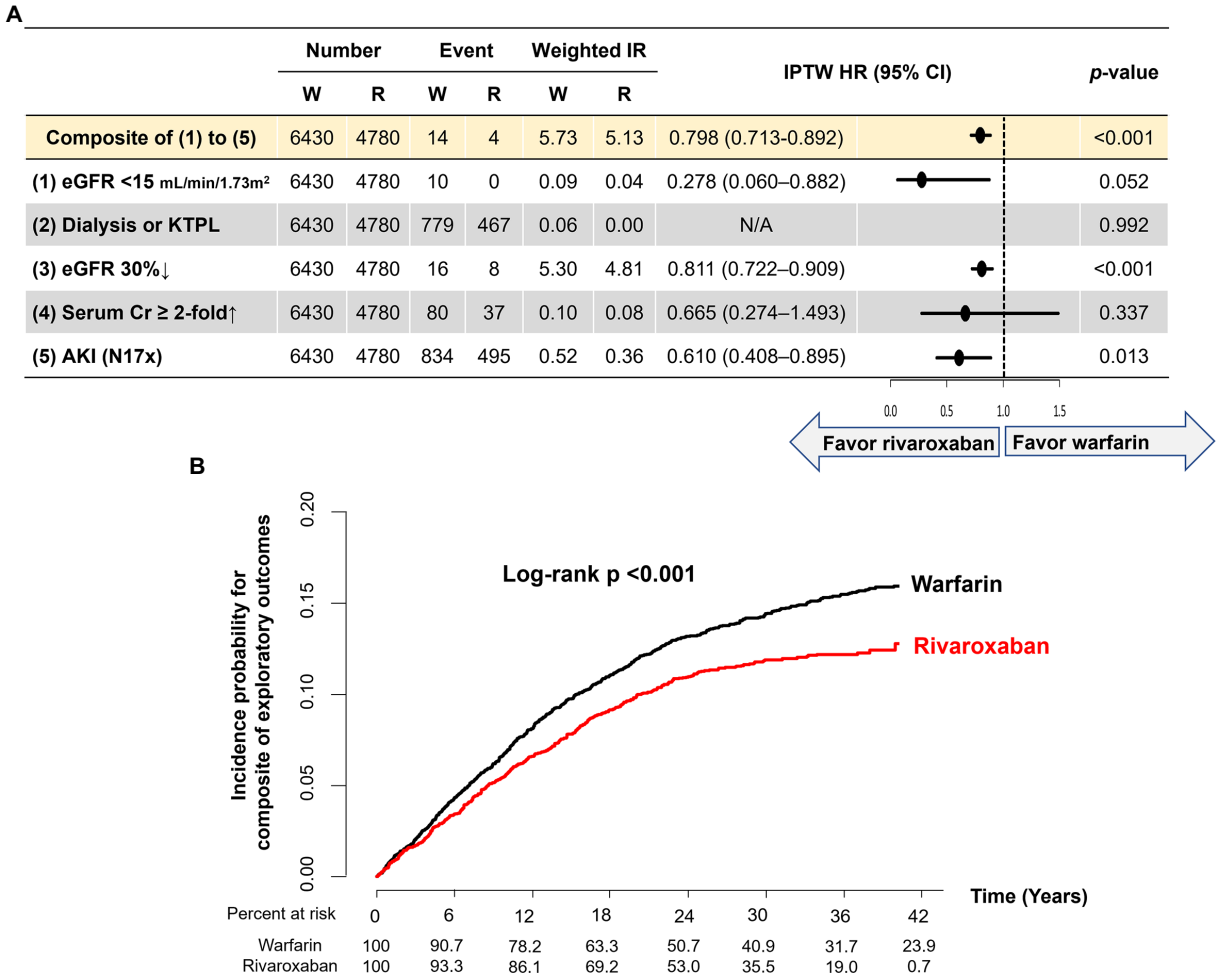
Exploratory analysis in patients with baseline and follow-up eGFR measurements. **(A)**. Weighted event numbers, incidence rates, and hazard ratios for five renal outcomes and composite of renal outcomes between rivaroxaban and warfarin groups **(B)**. Weighted Kaplan–Meier curves for the composite of renal outcomes between rivaroxaban and warfarin groups. Incidence rate, per 100 person-years. AKI, acute kidney injury; CI, confidence interval; Cr, creatinine; eGFR, estimated glomerular filtration rate; HR, hazard ratio; KTPL, kidney transplantation; IPTW, inverse probability of treatment weighting; IR, incidence rate; R, rivaroxaban; W, warfarin.

During a median follow-up of 2.28 (IQR 1.42–3.19) years, five renal outcomes and the composite of renal outcomes were evaluated in the two groups. Weighted event numbers, incidence rates, and HRs are shown in Figure 4A. Compared with warfarin, the rivaroxaban group was associated with significant 72, 20 and 39% reductions in the risks of developing eGFR lower than 15 ml/min/1.73 m^2^ at follow-up measurement, 30% decline in eGFR, and incidence of AKI, respectively. Although there was no statistically significant difference in the risk of serum creatinine doubling, the rivaroxaban group had a lower chance than the warfarin group. During the follow-up period, none of the patients in this exploratory analysis started dialysis or had kidney transplantation. For the composite of five renal outcomes, the rivaroxaban group showed a lower risk than warfarin (HR 0.798, 95% CI 0.713–0.892, *p* < 0.001; Figures 4A,B).

## Discussion

In this large-scale observational cohort, we observed very consistent findings that rivaroxaban was associated with a lower risk of renal adverse outcomes than warfarin in Korean patients with AF. Also, consistently with the general consensus, we confirmed that rivaroxaban was associated with a lower risk of ischemic stroke, intracranial hemorrhage, and all-cause death than warfarin. The effect of rivaroxaban on renal preservation was more accentuated in patients with underlying renal function impairment. The strength of this study included a large number of patients with AF treated in diverse clinical practice settings who had linked insurance claims and laboratory results. Also, this analysis allowed us to examine multiple renal outcomes to evaluate the consistency of results across a variety of renal outcomes.

Patients with AF should be aware of the potential deterioration in renal function. Renal impairment puts individuals with AF at greater risk of thromboembolism and bleeding (22). Also, the dose of NOACs may need to be adjusted with renal function decline, or the prescription of NOACs should be discontinued if significant renal impairment develops (23). Since anticoagulation therapy should be continued throughout a patients’ entire life for those with AF, preserving renal function has become an important issue for optimal care in patients with AF. From the post-hoc analysis of the RE-LY trial, dabigatran, a direct thrombin inhibitor, firstly showed a protective effect from the progressive renal function decline compared with warfarin (4). Interestingly, warfarin with an increased international normalized ratio (INR) out of the therapeutic range showed a significantly rapid progression of renal function decline than dabigatran. In contrast, warfarin with mainly below therapeutic INR rage showed similar renal function decline to dabigatran (4). Considering poor INR control of Asians, mainly with lower INR than therapeutic ranges (13, 24), we needed additional Asian data to provide a comprehensive comparison of the risk of renal outcome caused by NOAC versus warfarin. Two previous reports from the Taiwanese population were based on the nationwide administrative claims database (6, 9). According to these studies, dabigatran, rivaroxaban, and apixaban were associated with a lower risk of AKI (6, 9). Although these studies included many patients, approximately 6,000–28,000 patients in each NOAC group, the study outcome was only defined by diagnostic codes of AKI without laboratory measurements. The present study, including many Asian patients, showed consistent findings with previous observational studies of non-Asians (5, 7, 8) and Asians (6, 9). Furthermore, in a subset of patients with laboratory results, we first confirmed that rivaroxaban benefited renal preservation in various definitions of renal outcomes in Asian patients with AF.

In previous studies, including three NOACs (rivaroxaban, dabigatran, and apixaban), the results were slightly different among studies (5, 9, 10). Compared with warfarin, rivaroxaban was associated with lower risks of a 30% decline in eGFR, doubling of serum creatinine, and AKI, but dabigatran was only associated with a 30% decline in eGFR and AKI, and apixaban did not show significant risk reduction for the any of the renal outcomes (5). With AKI defined by diagnostic codes, rivaroxaban and dabigatran were associated with a lower risk of AKI than warfarin, but apixaban showed comparable results with warfarin (10). In Asian patients with AF, all three NOACs showed a similar risk reduction of AKI defined by diagnostic codes to warfarin (9). NOACs’ renal preservation compared with warfarin is often attributed to warfarin’s hazardous effects, such as glomerular microhemorrhage, vascular inflammation, or calcification (4). Further studies are required to discover the difference among NOACs on the renal protection effect, especially edoxaban, and consider the dose–response relationship.

This study highlighted that rivaroxaban reduced the risk of renal failure in patients with CKD compared with those without. In the subgroup analyses, patients with underlying CKD and those with baseline eGFR ≤ 60 ml/min/1.73 m^2^ showed greater relative risk reduction with rivaroxaban than warfarin. Patients who are more vulnerable to the risk of kidney failure might get more benefit from rivaroxaban’s kidney protection effect. Kidney failure due to acute tubular injury with microhemorrhage might be more critical in patients with a smaller reservoir because of underlying renal impairment. This finding was consistently observed in previous studies (6, 7, 9). Careful selection of the anticoagulation agent and close follow-up of kidney function should be emphasized in this population. From Korean AF patients with mildly impaired renal function (creatinine clearance 50–60 ml/min), we previously reported that rivaroxaban 15 mg once daily was associated with a lower risk of ischemic stroke, intracranial hemorrhage, and hospitalization for major bleeding than warfarin. Additionally, rivaroxaban 15 mg once daily showed a comparable risk of ischemic stroke, intracranial hemorrhage, and hospitalization for major bleeding with rivaroxaban 20 mg once daily (25).

Recently, consistent results have been updated in various subsets of patients with elderly (26) and those with diabetes (7), and even a meta-analysis has been reported (27); thus, it is quite evident that NOAC is superior to warfarin for renal preservation. Our study supported its reasoning using data from large-scale Asian patients and laboratory data.

## Limitations

First, despite careful adjustment using IPTW, our study may still be subject to residual confounding. In database analysis where randomization is not possible, such PS-based methods as matching or IPTW serve to harmonize comparison groups concerning patient characteristics. However, residual confounding was caused by unmeasured factors such as laboratory values (e.g., time in the therapeutic window for warfarin), missing data, miscoding, or tactical coding issues. Second, the application of both on-treatment and ITT analysis in non-randomized studies has different limitations as follows: an on-treatment method leads to a loss of information on the reasons for treatment discontinuation, while an ITT approach would not reflect changes on treatments affecting the primary outcome. In our study, the primary purpose of this study was to compare warfarin and rivaroxaban for the risk of kidney failure in anticoagulated patients with AF. In real-world clinical practice, many patients changed their OAC agents from warfarin to NOAC (28). The clinical impact of warfarin might widely mix with various NOACs in patients who changed their OAC agents from warfarin to NOAC in ITT analysis. Therefore, we believe it is more appropriate for the main analysis to be an on-treatment manner rather than ITT manner. Furthermore, we analyzed an ITT analysis for a sensitivity analysis. Although there was a slight attenuation on the HRs, the results were largely consistent with the main analysis in an on-treatment manner. Third, to control the possible effect of prior use of warfarin, we only include OAC new users from 1 January 2014. This could result in an overall short-term follow-up duration for both groups. Fourth, in the present study, we did not perform a comprehensive comparison among different NOACs for the risk of kidney failure because of the limitation of dataset. Comparative analysis among DOACs on the risk of kidney failure might be a valuable topic foe patient care. Further research is needed to elucidate the relative risk difference of different NOACs on the risk of kidney failure compared to warfarin or NOACs. Fifth, because of an inherent limitation of the data source, we could not analyze the treatment quality of warfarin using the time in therapeutic range of INR. Furthermore, the results can be generalized only to Korean patients with AF. Informative censoring might exist in patients who discontinued the index treatment. This was evaluated by a sensitivity analysis that follows the ITT approach.

## Conclusion

In Korean patients with AF, rivaroxaban was associated with a lower risk of renal adverse outcomes than warfarin. The renal preservation effect of rivaroxaban compared with warfarin was particularly pronounced in patients with preexisting renal impairment. Rivaroxaban should be explored for anticoagulation therapy in AF patients at high risk of renal function decline.

## Data availability statement

Publicly available datasets were analyzed in this study. This data can be found at: http://nhiss.nhis.or.kr/bd/ab/bada000eng.do.

## Ethics statement

Ethical review and approval was not required for the study on human participants in accordance with the local legislation and institutional requirements. Written informed consent for participation was not required for this study in accordance with the national legislation and the institutional requirements.

## Author contributions

S-RL contributed to the design of the study, interpretation of the results and prepared the manuscript. E-KC, SO, KA, and GL contributed to the design of the study, interpretation of the results and critical revision of the manuscript. S-HP and K-DH contributed to the analysis of data and interpretation of results. All authors contributed to the article and approved the submitted version.

## Conflict of interest

E-KC: Research grants or speaking fees from Bayer, Biosense Webster, BMS/Pfizer, Chong Kun Dang, Daiichi Sankyo, Dreamtech Co., Ltd., Jeil Pharmaceutical Co. Ltd., Medtronic, Samjinpharm, Seers Technology, and Skylabs. GL: Consultant and speaker for BMS/Pfizer, Boehringer Ingelheim, and Daiichi Sankyo. No fees are received personally. KA was employed by Bayer AG. This study was funded by Bayer AG. Bayer AG contributed to the design and conduct of the study; management and interpretation of the data; preparation, review, and approval of the manuscript; and the decision to submit the manuscript for publication.

## Publisher’s note

All claims expressed in this article are solely those of the authors and do not necessarily represent those of their affiliated organizations, or those of the publisher, the editors and the reviewers. Any product that may be evaluated in this article, or claim that may be made by its manufacturer, is not guaranteed or endorsed by the publisher.
