# Supplementary material for: Renal outcomes of rivaroxaban compared with warfarin in Asian patients with nonvalvular atrial fibrillation: A nationwide population-based cohort study
Source: Front Cardiovasc Med. 2023 Feb 23;10:1040834. doi: 10.3389/fcvm.2023.1040834 (PMC9996329; doi:10.3389/fcvm.2023.1040834)
Supplement: Supplementary file 1 [file Data_Sheet_1.docx]

**Supplementary Materials**

**Supplementary Methods**

**Data sources**

**Representativeness**

**Supplementary Results**

**Sensitivity analyses**

**Supplementary Tables**

**Supplementary Table 1. Definitions of covariates and outcomes**

**Supplementary Table 2. Charlson Comorbidity Index**

**Supplementary Table 3. Baseline characteristics of total, warfarin, and rivaroxaban groups**

**Supplementary Table 4. Crude event numbers, incidence rates, and unadjusted hazard ratios for primary and secondary outcomes (on-treatment analysis set)**

**Supplementary Table 5. Multivariable Cox analysis based on the on-treatment analysis set excluding CHA_2_DS_2_-VASc score, CCI, or both of these in the final model**

**Supplementary Table 6. Baseline characteristics of exploratory analysis set (patients who had baseline and follow-up national health examination results), before and after IPTW**

**Supplementary Figure**

**Supplementary Figure 1. Distribution of propensity scores in warfarin and rivaroxaban groups after IPTW (main analysis set)**

**Supplementary Figure 2. Various sensitivity analyses for the primary outcome (kidney failure)**

**Supplementary Methods**

*Data sources*

This retrospective observational nationwide cohort study was conducted using administrative claims data of the Korean National Health Insurance Service (NHIS) and the National Health Insurance Cooperation (NHIC) linked health check-up database between 2013 and 2018. The Korean NHIS provides comprehensive medical care coverage for the entire Korean population (approximately 50 million people). Those insured by NHI pay monthly insurance contributions for medical services provided by health care providers. The medical aid program covers low-income households with a minimum livelihood.

The NHI program managed by NHIS, Medical Aid for low-income groups, and long-term care insurance cover 100% of the Korean population, approximately 50 million in 2019. Since the NHIS pays costs based on the billing records of health care providers, the NHIS created a database to collect the required information on insurance eligibility, insurance contributions, medical history, and medical institutions. The NHI program provides health care benefits, including diagnosis, laboratory tests, drugs, medical materials, treatment, surgery, rehabilitation, hospitalization, nursing, transportation by healthcare providers, and health screening services.

The Korean NHIS database includes individual demographic information and all data related to medical expenses, including diagnoses based on the International Classification of Diseases 10^th^ Clinical Modification codes, prescription, and procedure records based on The Electronic Data Interchange (EDI) medical procedure code (1, 2). The NHI provides regular health check-ups and a cancer screening program to discover and treat the disease early and improve health. All insured Koreans, at least 40 years old, and their dependents can receive biannual health checks without cost (1). A national health check-up provided by the Korean NHIC is conducted biennially. It includes physical examinations of the patients, regular blood tests, chest X-ray examinations, and questionnaires on their medical history. The national health screening database incorporates detailed lifestyle questionnaires, laboratory results, and anthropometric measurements. Health screening programs comprise general national health screening, health screening programs for transitional ages (40 and 66 years), early childhood health screening programs, and cancer screening programs (3). All insured Koreans aged 40 years or older and their dependents are recommended to undergo biannual general health screening without cost. The participation rate of the general health screening program among eligible participants was 74.8% in 2014 (3). The national health screening database provides the estimated glomerular filtration rate or serum creatinine level.

The NHIS database has several strengths. First, since the NHIS database constitutes data for more than 52 million people, it is one of the largest claims datasets. The NHIS database also contains data regarding all age groups and the entire region, reducing selection bias. Moreover, the NHIS database is different from the Medicare and Medicaid program of the United States, which are restricted to either elders or those with low income. Second, the NHIS database includes health screening information containing detailed lifestyle questionnaires, laboratory results, and anthropometric measurements not included in other claims databases. Third, since the NHIS database has been linked to other datasets, such as mortality data from Statistics Korea, further detailed information is available for analysis. However, regulations and social consensus are needed to solve this issue.

The NHIS database also has several limitations. First, since this dataset was established for recording claims and reimbursements, the dataset may not be optimal for research purposes. There may be a discrepancy between the real disease and the diagnosis claimed by the healthcare providers. Over- or under-diagnosis may pose another limitation. Previous studies showed that approximately 70% of primary diagnosis codes concurred with medical records. This study used validated definitions for outcomes and covariates whenever available. Second, since not all eligible subjects undergo regular health screening, the NHIS database may include data based on only parts of the population. Third, the NHIS database contains basic laboratory test results, thus, excluding more detailed test results, such as time in therapeutic range in warfarin users. Fourth, the NHIS database does not include information about healthcare services not covered by the NHI system, such as cosmetic procedures, over-the-counter medicines, and uninsured new drugs or treatment.

*Representativeness*

The study population would represent Korean patients with AF who were prescribed anticoagulation therapy. The study is conducted using administrative claims data of the Korean NHIS, which provides comprehensive medical care coverage for the entire Korean population (approximately 50 million people). The Korean NHIS provides a randomly selected 50% sample cohort for the analysis. NHIS provides a database that includes health information data collected, managed, and maintained by the National Health Insurance Corporation and can be customized as requested for the policy and academic research. NHIS provides a sample research database representing a sample cohort that NHIS defines for a specified period. The NHIS database has an extremely large size; usually, 20–50% of the entire data are randomly selected due to the limited capacity of data provision. Therefore, the NHIS database provides a representative sample of Korean patients with AF.

*Statistical Methods*

*Inverse probability of treatment weighting [IPTW]*

Weighting by the inverse probability of treatment results in an artificial population of the synthetic sample, in which treatment assignment is independent of measured baseline characteristics. This analysis deployed IPTW with stabilized weights, which ensure more robust effect estimates. The use of stabilized weights in the synthetic data preserves the sample size of the original data. It provides an appropriate estimate of the treatment effect from the conventional regression with SWs (4). By applying the IPTW method using, the propensity score assessment needs to be done, whether the weighting procedure succeeded in balancing patient characteristics between treatment groups. We assessed the balance by using absolute standardized differences (ASDs). The distributions of propensity scores and stabilized weights will be inspected for initial and synthetic samples. An ASD of 0.1 or less was considered a negligible difference between the two groups. The initial propensity score model will be adapted in case of remaining imbalance.

**References**

1. Choi EK. Cardiovascular Research Using the Korean National Health Information Database. *Korean circulation journal.* (2020) 50:754-72. doi: 10.4070/kcj.2020.0171.

2. Song SO, Jung CH, Song YD, Park CY, Kwon HS, Cha BS, et al. Background and Data Configuration Process of a Nationwide Population-Based Study Using the Korean National Health Insurance System. *Diabetes Metab J.* (2014) 38:395-403. doi: 10.4093/dmj.2014.38.5.395.

3. Seong SC, Kim YY, Khang YH, Park JH, Kang HJ, Lee H, et al. Data Resource Profile: The National Health Information Database of the National Health Insurance Service in South Korea. *Int J Epidemiol.* (2017) 43:799-800. doi: 10.1093/ije/dyw253.

4. Xu S, Ross C, Raebel MA, Shetterly S, Blanchette C, Smith D. Use of Stabilized Inverse Propensity Scores as Weights to Directly Estimate Relative Risk and Its Confidence Intervals. *Value Health.* (2010) 13:273-7. doi: 10.1111/j.1524-4733.2009.00671.x.

**Supplementary Results**

*Sensitivity analyses*

For the primary outcome we performed various sensitivity analyses that demonstrated results consistent with the main analysis. Rivaroxaban was associated with significant reductions in the risk for kidney failure in all analyses, with HRs ranging from 0.323 to 0.625 (**Supplementary Figure 2)**. In the ITT study set, the median follow-up duration was 2.84 (IQR 1.72–3.85) years; the warfarin group had a longer follow-up duration than rivaroxaban (3.54 [2.06–4.22] vs 2.22 [1.45–2.93], *p*<0.001). After IPTW following the ITT approach, HR for kidney failure was slightly more attenuated than the on-treatment approach. However, the rivaroxaban group was associated with a significantly lower risk of kidney failure than the warfarin group (HR 0.625, 95% CI 0.520–0.746).

Results of multivariable-adjusted Cox analyses in both the on-treatment (HR 0.323, 95% CI 0.249–0.420) and ITT analyses (HR 0.516, 95% CI 0.427–0.624) were consistent with IPTW. In the analysis using IPTW with 5% trimming, the rivaroxaban group was associated with a lower risk of kidney failure than the warfarin group in both the on-treatment (HR 0.323, 95% CI 0.240–0.427) and ITT (HR 0.533, 95% CI 0.434–0.651) populations, as shown in the main analysis. When we excluded the CHA_2_DS_2_-VASc score, CCI, or both of these in the final model, the HRs were consistent with the main analysis (**Supplementary Table** **5**).

In the sensitivity analyses with a 6-month lag period or with restriction of follow-up within 12 months, the results were consistent with the main analysis (HRs 0.422, 95% CI 0.294–0.605 and 0.348, 95% CI 0.247–0.480, respectively).

Patients with baseline eGFR measurements comprised 32,336 (67.4%) of the total study population. In these patients, we added the baseline eGFR value in the multivariable Cox analysis. Even after accounting for baseline eGFR, the rivaroxaban group had a consistently lower risk of kidney failure than the warfarin group (HR 0.545, 95% CI 0.391-0.759, p<0.001, **Supplementary Figure 2**).

**Supplementary Table 1. Definitions of covariates and outcomes**

| **Diagnosis** | **ICD-10-CM code and definition** | **Diagnostic definition** |
| --- | --- | --- |
| **Inclusion/exclusion criteria** | | |
| **Atrial fibrillation** | I48.0-I48.4, I48.9 | Admission or outpatient department ≥1 |
| **Valvular atrial fibrillation** | I05.0, I05.2, I05.9, Z95.2-Z95.4 | Admission or outpatient department ≥1 |
| **Pulmonary embolism** | I26 | Admission ≥1 |
| **Deep vein thrombosis** | I80.2 | Admission ≥1 |
| **Received joint replacement operation** | N0711, N1711, N1721, N2070, N3710, N3721, N3717, N3720, N2072, N2077, N3722, N3727 | Admission ≥1 |
| **End stage renal disease** | N18.5, Z49 | Dialysis ≥2  Dialysis: hemodialysis (O7011-O7020), or peritoneal dialysis (O7017, O7075) |
| **Comorbidities** | | |
| **Hypertension** | I10-I13, I15; and minimum 1 prescription of anti-hypertensive drug (thiazide, loop diuretics, aldosterone antagonist, alpha-/beta-blocker, calcium-channel blocker, angiotensin-converting enzyme inhibitor, angiotensin II receptor blocker). | Admission ≥1 or outpatient department ≥2 |
| **Diabetes** | E11-E14; and minimum 1 prescription of anti-diabetic drugs (sulfonylureas, metformin, meglitinides, thiazolidinediones, dipeptidyl peptidase-4 inhibitors, α-glucosidase inhibitors, and insulin). | Admission ≥1 or outpatient department ≥2 |
| **Dyslipidemia** | E78 | Admission or outpatient department ≥1 |
| **Heart failure** | I50 | Admission or outpatient department ≥1 |
| **Ischemic stroke** | I63, I64 | Admission or outpatient department ≥1 |
| **Myocardial infarction** | I21, I22 | Admission or outpatient department ≥1 |
| **Peripheral artery disease** | I70, I73 | Admission or outpatient department ≥2 |
| **Chronic kidney disease** | N18, N19 | Admission or outpatient department ≥1 |
| **Chronic obstructive pulmonary disease** | J41-44 | Admission ≥1 |
| **Cancer** | C00-97 and RID code (V193) | Admission or outpatient department ≥1 |
| **Scores** | | |
| **CHA_2_DS_2_-VASc score** | Heart failure (1 point), hypertension (1 point), age ≥75 years (2 points), diabetes (1 point), previous stroke/systemic embolism/transient ischemic attack (2 points), vascular disease (prior myocardial infarction or peripheral artery disease, 1 point) and female sex (1 point) | |
| **Charlson Comorbidity Index** | Supplementary Table 2 | |
| **Study outcomes of the main analysis** | | |
| **Primary outcome: Kidney failure** | Kidney transplantation (R3280) or hemodialysis (O7011-O7020), or peritoneal dialysis (O7017, O7075) | Dialysis ≥2 |
| **Secondary outcomes** | | |
| **Ischemic stroke** | I63, I64 | Primary diagnosis, admission ≥1 (≥3 days), and brain imaging (CT or MRI) ≥1 |
| **Intracranial hemorrhage** | I60-62 | Primary diagnosis, admission ≥1 (≥3 days), and brain imaging (CT or MRI) ≥1 |
| **Major gastrointestinal bleeding** | I85, K22.1, I22.8, K25.0, K25.2, K25.4, K25.6, K26.0, K26.2, K26.4, K26.6, K27.0, K27.2, K27.4, K27.6, K28.0, K28.2, K28.4, K28.6, K29.0, K31.8, K92.0, K92.1, K92.2, K55.2, K57.0, K57.1, K57.2, K57.3, K57.4, K57.5, K57.8, K57.9, K62.5, K66.1 | Primary diagnosis, admission ≥1 |
| **Major bleeding** | Intracranial hemorrhage + major gastrointestinal bleeding | Each definition was described as above. |
| **All-cause death** | Death from any cause | Data from Statistics Korea linked with the Korean NHIS database. |
| **Study outcomes of exploratory analysis** | | |
| **(1) eGFR <15 ml/min/1.73m^2^ at follow-up measurement** | |  |
| **(2) Dialysis or kidney transplantation** | Kidney transplantation (R3280) or hemodialysis (O7011-O7020), or peritoneal dialysis (O7017, O7075) | Dialysis ≥2 |
| **(3) ≥ 30% decline in eGFR** | Comparison between baseline and follow-up measurements | |
| **(4) Doubling of serum creatinine** | Comparison between baseline and follow-up measurements | |
| **(5) Acute kidney injury** | N17x | Emergency department visit or hospitalization with a diagnostic code of AKI |
| **Composite of five renal outcomes** | (1) + (2) + (3) + (4) + (5), the first occurrence among any renal outcomes | Each definition was described as above. |

Abbreviations: AKI, acute kidney injury; CHA_2_DS_2_-VASc, congestive heart failure, hypertension, age ≥75 (doubled), diabetes mellitus, prior stroke or transient ischaemic attack (doubled), vascular disease, age 65–74, female; CT, computed tomography; eGFR, estimated glomerular filtration rate; ICD-10-CM, International Classification of Diseases 10^th^ Clinical Modification; MRI, magnetic resonance image; NHIS, national health insurance service.

**Supplementary Table 2. Charlson Comorbidity Index**

| **Category** | **Weights** | **Disease** | **ICD-10-CM code** |
| --- | --- | --- | --- |
| **Myocardial infarction** | 1 | Acute myocardial infarction | I21 |
|  |  | Subsequent myocardial infarction | I22 |
| **Congestive heart failure** | 1 | Heart failure | I50 |
| **Peripheral vascular disease** | 1 | Atherosclerosis | I70 |
|  |  | Other peripheral vascular disease | I73 |
| **Cerebrovascular disease** | 1 | Transient cerebral ischemic attacks and related syndromes | G45 |
|  |  | Vascular syndromes of brain in cerebrovascular diseases | G46 |
|  |  | Retinal vascular occlusion | H34 |
|  |  | Cerebrovascular disease | I60-I69 |
| **Dementia** | 1 | Dementia in Alzheimer disease | F00 |
|  |  | Vascular dementia | F01 |
|  |  | Dementia in other disease classified elsewhere | F02 |
|  |  | Unspecified dementia | F03 |
| **Chronic pulmonary disease** | 1 | Chronic lower respiratory diseases | J40-J47 |
|  |  | Lung disease due to external agents | J60-J67 |
| **Rheumatic disease**  **(connective tissue disorder)** | 1 | Rheumatoid arthritis with rheumatoid factor | M05 |
|  |  | Felty's syndrome | M05.0 |
|  |  | Rheumatoid lung disease with rheumatoid arthritis | M05.1 |
|  |  | Rheumatoid vasculitis with rheumatoid arthritis | M05.2 |
|  |  | Rheumatoid heart disease with rheumatoid arthritis | M05.3 |
|  |  | Rheumatoid myopathy with rheumatoid arthritis | M05.4 |
|  |  | Rheumatoid polyneuropathy with rheumatoid arthritis | M05.5 |
|  |  | Rheumatoid arthritis with involvement of other organs and systems | M05.6 |
|  |  | Rheumatoid arthritis with rheumatoid factor without organ or systems involvement | M05.7 |
|  |  | Other rheumatoid arthritis with rheumatoid factor | M05.8 |
|  |  | Rheumatoid arthritis without rheumatoid factor | M05.9 |
|  |  | Adult-onset Still's disease | M06.1 |
|  |  | Rheumatoid bursitis | M06.2 |
|  |  | Rheumatoid nodule | M06.3 |
|  |  | Inflammatory polyarthropathy | M06.4 |
|  |  | Other specified rheumatoid arthritis | M06.8 |
|  |  | Rheumatoid arthritis, unspecified | M06.9 |
|  |  | Giant cell arteritis with polymyalgia rheumatica | M31.5 |
|  |  | Systemic lupus erythematosus (SLE) | M32 |
|  |  | Drug-induced SLE | M32.0 |
|  |  | SLE with organ or system involvement | M32.1 |
|  |  | Other forms of SLE | M32.8 |
|  |  | SLE, unspecified | M32.9 |
|  |  | Dermatopolymyositis | M33 |
|  |  | Juvenile dermatomyositis | M33.0 |
|  |  | Other dermatomyositis | M33.1 |
|  |  | Polymyositis | M33.2 |
|  |  | Dermatopolymyositis, unspecified | M33.9 |
|  |  | Systemic sclerosis [scleroderma] | M34 |
|  |  | Progressive systemic sclerosis | M34.0 |
|  |  | CR(E)ST syndrome | M34.1 |
|  |  | Systemic sclerosis induced by drug and chemical | M34.2 |
|  |  | Other forms of systemic sclerosis | M34.8 |
|  |  | Systemic sclerosis, unspecified | M34.9 |
|  |  | Other overlap syndromes | M35.1 |
|  |  | Polymyalgia rheumatica | M35.3 |
|  |  | Dermato(poly)myositis in neoplastic disease | M36.0 |
| **Peptic ulcer disease** | 1 | Gastric ulcer | K25 |
|  |  | Duodenal ulcer | K26 |
|  |  | Peptic ulcer, site unspecified | K27 |
|  |  | Gastrojejunal ulcer | K28 |
| **Mild liver disease** | 1 | Chronic viral hepatitis | B18 |
|  |  | Alcoholic fatty liver | K70.0- K70.3, K70.9 |
|  |  | Alcoholic hepatitis |  |
|  |  | Alcoholic fibrosis and sclerosis of liver |  |
|  |  | Alcoholic cirrhosis of liver |  |
|  |  | Alcoholic liver disease, unspecified |  |
|  |  | Toxic liver disease with chronic persistent hepatitis | K71.3- K71.5, K71.7 |
|  |  | Toxic liver disease with chronic lobular hepatitis |  |
|  |  | Toxic liver disease with chronic active hepatitis |  |
|  |  | Toxic liver disease with fibrosis and cirrhosis of liver |  |
|  |  | Chronic hepatitis, not elsewhere classified | K73 |
|  |  | Fibrosis and cirrhosis of liver | K74 |
|  |  | Fatty (change of) liver, not elsewhere classified | K76.0-K76.4, K76.8, K76.9 |
|  |  | Nonalcoholic fatty liver disease |  |
|  |  | Central hemorrhagic necrosis of liver |  |
|  |  | Infarction of liver |  |
|  |  | Hepatic angiomatosis |  |
|  |  | Other specified disease of liver |  |
|  |  | Simple cyst of liver |  |
|  |  | Focal nodular hyperplasia of liver |  |
|  |  | Hepatoptosis |  |
|  |  | Liver disease, unspecified |  |
|  |  | Liver transplant status | Z94.4 |
| **Diabetes without chronic**  **complication** | 1 | with coma | E10.0, 10.1, 10.6, 10.8, 10.9 |
|  |  | with ketoacidosis | E11.0, 11.1, 11.6, 11.8, 11.9 |
|  |  | with other specified complications | E12.0, 12.1, 12.6, 12.8, 12.9 |
|  |  | with unspecified complications | E13.0, 13.1, 13.6, 13.8, 13.9 |
|  |  | without complications | E14.0, 14.1, 14.6, 14.8, 14.9 |
| **Diabetes with chronic**  **complication** | 2 | with renal complications | E10.2, 10.3, 10.4, 10.5, 10.7 |
|  |  | with ophthalmic complications | E11.2, 11.3, 11.4, 11.5, 11.7 |
|  |  | with neurologic complications | E12.2, 12.3, 12.4, 12.5, 12.7 |
|  |  | with peripheral circulatory complications | E13.2, 13.3, 13.4, 13.5, 13.7 |
|  |  | with multiple complications | E14.2, 14.3, 14.4, 14.5, 14.7 |
| **Hemi/paraplegia** | 2 | Tropical spastic paraplegia | G04.1 |
|  |  | Hereditary spastic paraplegia | G11.4 |
|  |  | Spastic quadriplegic cerebral palsy | G80.0 |
|  |  | Spastic diplegic cerebral palsy | G80.1 |
|  |  | Spastic hemiplegic cerebral palsy | G80.2 |
|  |  | Flaccid hemiplegia | G81.0 |
|  |  | Spastic hemiplegia | G81.1 |
|  |  | Hemiplegia, unspecified | G81.9 |
|  |  | Flaccid paraplegia | G82.0 |
|  |  | Spastic paraplegia | G82.1 |
|  |  | Paraplegia, unspecified | G82.2 |
|  |  | Flaccid tetraplegia | G82.3 |
|  |  | Spastic tetraplegia | G82.4 |
|  |  | Tetraplegia, unspecified | G82.5 |
|  |  | Diplegia of upper limbs | G83.0 |
|  |  | Paralytic syndrome, unspecified | G83.9 |
| **Renal disease** | 2 | Hypertensive renal disease | I12 |
|  |  | Hypertensive heart and renal disease with renal failure | I13.1 |
|  |  | Chronic nephritic syndrome | N03 |
|  |  | Unspecified nephritic syndrome | N05 |
|  |  | Chronic kidney disease | N18 |
|  |  | Unspecified kidney failure | N19 |
|  |  | Disorders resulting from impaired renal tubular function | N25 |
|  |  | Care involving dialysis | Z49 |
|  |  | Transplanted organ and tissue status - kidney | Z94.0 |
|  |  | Dependence on renal dialysis | Z99.2 |
| **Cancer** | 2 | Any tumor, malignant neoplasm | C00-76, C97 |
|  |  | Any tumor, in situ neoplasm | D00-09 |
|  |  | Any tumor, Benign neoplasm | D10-36 |
|  |  | Any tumor, Neoplasm of unknown behavior | D37-48 |
|  |  | Leukemia | C91-95 |
|  |  | Lymphoma | C81-86 |
| **Metastatic cancer** | 3 | Metastatic solid tumor | C77-80 |
| **Moderate to severe**  **liver disease** | 3 | Esophageal varices | I85 |
|  |  | Gastric varices | I86.4 |
|  |  | Esophageal varices without bleeding in diseases classified elsewhere | I98.2 |
|  |  | Alcoholic hepatic failure | K70.4 |
|  |  | Toxic liver disease with hepatic necrosis | K71.1 |
|  |  | Hepatic failure (acute/chronic) due to drugs |  |
|  |  | Chronic hepatic failure | K72.1, K72.9 |
|  |  | Hepatic failure, unspecified |  |
|  |  | Hepatic veno-occlusive disease | K76.5-K76.7 |
|  |  | Portal hypertension |  |
|  |  | Hepatorenal syndrome |  |
| **Human immunodeficiency**  **Virus (HIV)** | 6 | HIV disease resulting in infectious and parasitic diseases | B20 |
|  |  | HIV disease resulting in malignant neoplasm | B21 |
|  |  | HIV disease resulting in other specified diseases | B22 |
|  |  | HIV disease resulting in other conditions | B23 |

**Supplementary Table 3. Baseline characteristics of total study population, warfarin group and rivaroxaban group**

|  | **Total** | **Warfarin** | **Rivaroxaban** | **p-value** |
| --- | --- | --- | --- | --- |
| **n** | 47,946 | 30,933 | 17,013 |  |
| **Age, years** | 70.1±11.7 | 69.0±12.3 | 72.1±10.1 | <0.001 |
| **<65 years** | 13,412 (28.0) | 9944 (32.2) | 3468 (20.4) | <0.001 |
| **65 to <75 years** | 15,386 (32.1) | 9412 (30.4) | 5974 (35.1) |  |
| **≥75 years** | 19,148 (39.9) | 11,577 (37.4) | 7571 (44.5) |  |
| **Sex, men** | 27,865 (58.1) | 18,260 (59.0) | 9605 (56.5) | <0.001 |
| **CHA_2_DS_2_-VASc** | 3.9±1.9 | 3.8±2.0 | 4.1±1.7 | <0.001 |
| **CHA_2_DS_2_-VASc ≥3** | 36,273 (75.7) | 22,494 (72.7) | 13,779 (81.0) | <0.001 |
| **Charlson Comorbidity Index** | 4.0±2.5 | 4.0±2.5 | 4.0±2.4 | 0.522 |
| **Charlson Comorbidity Index ≥3** | 33,422 (69.7) | 21,444 (69.3) | 11,978 (70.4) | 0.013 |
| **Hypertension** | 39,605 (82.6) | 25,023 (80.9) | 14,582 (85.7) | <0.001 |
| **Diabetes** | 12,684 (26.5) | 8067 (26.1) | 4617 (27.1) | 0.011 |
| **Dyslipidemia** | 25,647 (53.5) | 16,290 (52.7) | 9357 (55.0) | <0.001 |
| **Heart failure** | 20,142 (42.0) | 12,550 (40.6) | 7592 (44.6) | <0.001 |
| **Prior stroke** | 13,826 (28.8) | 9511 (30.8) | 4315 (25.4) | <0.001 |
| **Prior myocardial infarction** | 3030 (6.3) | 2026 (6.6) | 1004 (5.9) | 0.005 |
| **Peripheral artery disease** | 11,303 (23.6) | 6948 (22.5) | 4355 (25.6) | <0.001 |
| **Chronic kidney disease** | 2623 (5.5) | 1899 (6.1) | 724 (4.3) | <0.001 |
| **COPD** | 4347 (9.1) | 2975 (9.6) | 1372 (8.1) | <0.001 |
| **Cancer** | 3371 (7.0) | 2003 (6.5) | 1368 (8.0) | <0.001 |
| **Antiplatelet use** |  |  |  |  |
| **None** | 31,469 (65.5) | 18,790 (60.7) | 12,679 (74.5) | <0.001 |
| **Aspirin only** | 8699 (18.1) | 6562 (21.2) | 2137 (12.6) |  |
| **P2Y12 only** | 2782 (5.8) | 1889 (6.1) | 902 (5.3) |  |
| **both** | 4996 (10.4) | 3701 (12.0) | 1295 (7.6) |  |
| **Rivaroxaban dose** |  |  |  |  |
| **20 mg once daily** | N/A | N/A | 8354 (49.1) |  |
| **15 mg once daily** | N/A | N/A | 8659 (50.9) |  |

Continuous variables are shown as mean and standard deviation. Categorical variables are presented as numbers (percentages).

Abbreviations: COPD, chronic obstructive pulmonary disease; N/A, not available.

**Supplementary Table 4. Crude event numbers, incidence rates, and unadjusted hazard ratios for primary and secondary outcomes (on-treatment analysis set)**

|  | **Treatment** | **Number** | **Event** | **IR** | **Unadjusted HR**  **(95% CI)** | ***p*-value** |
| --- | --- | --- | --- | --- | --- | --- |
| **Kidney failure** | Warfarin | 30,933 | 342 | 0.89 | 1 (reference) | <0.001 |
|  | Rivaroxaban | 17,013 | 68 | 0.29 | 0.323 (0.249-0.420) |  |
| **Ischemic stroke** | Warfarin | 30,933 | 1052 | 2.78 | 1 (reference) | <0.001 |
|  | Rivaroxaban | 17,013 | 465 | 1.97 | 0.745 (0.667-0.831) |  |
| **ICH** | Warfarin | 30,933 | 232 | 0.60 | 1 (reference) | 0.002 |
|  | Rivaroxaban | 17,013 | 100 | 0.42 | 0.690 (0.545-0.873) |  |
| **Major GIB** | Warfarin | 30,933 | 395 | 1.03 | 1 (reference) | 0.144 |
|  | Rivaroxaban | 17,013 | 265 | 1.12 | 1.125 (0.961-1.318) |  |
| **Major bleeding** | Warfarin | 30,933 | 768 | 2.01 | 1 (reference) | 0.835 |
|  | Rivaroxaban | 17,013 | 464 | 1.97 | 0.988 (0.879-1.11) |  |
| **All-cause death** | Warfarin | 30,933 | 2227 | 5.76 | 1 (reference) | <0.001 |
|  | Rivaroxaban | 17,013 | 1165 | 4.88 | 0.785 (0.815-0.940) |  |

Incidence rate, per 100 person-years

Abbreviations: CI, confidence interval; GIB, gastrointestinal bleeding; HR, hazard ratio; ICH, intracranial hemorrhage; IR, incidence rate.

**Supplementary Table 5. Multivariable Cox analysis based on the on-treatment analysis set excluding CHA_2_DS_2_-VASc score, CCI, or both of these in the final model**

|  | Adjusted hazard ratio  (95% confidence interval) | p-value |
| --- | --- | --- |
| Main analysis (IPTW OT) | 0.389 (0.300-0.499) | <0.001 |
| Multivariable adjusted Cox (on-treatment) | 0.409 (0.313-0.534) | <0.001 |
| Excluding CHA_2_DS_2_-VASc score | 0.411 (0.315-0.537) | <0.001 |
| Excluding CCI | 0.408 (0.313-0.533) | <0.001 |
| Excluding both | 0.411 (0.315-0.537) | <0.001 |

Abbreviation: CCI, Charlson comorbidity index.

**Supplementary Table 6. Baseline characteristics of exploratory analysis set (patients who had baseline and follow-up national health examination results), before and after IPTW**

|  |  | **Before IPTW** | | | **After IPTW** | | |
| --- | --- | --- | --- | --- | --- | --- | --- |
|  | **Total** | **Warfarin** | **Rivaroxaban** | **ASD** | **Warfarin** | **Rivaroxaban** | **ASD** |
| **n** | 11,210 | 6430 | 4780 |  | 6453 | 4745 |  |
| **Age, years** | 67.0±9.7 | 65.6±10.4 | 69.0±8.3 | 0.360 | 67.1±9.9 | 67.3±9.2 | 0.021 |
| **<65 years** | 3977 (35.5) | 2706 (42.1) | 1271 (26.6) |  | 2299 (35.6) | 1598 (33.7) |  |
| **65 to <75 years** | 4649 (41.5) | 2406 (37.4) | 2243 (46.9) |  | 2625 (40.7) | 2057 (43.4) |  |
| **≥75 years** | 2584 (23.1) | 1318 (20.5) | 1266 (26.5) |  | 1530 (23.7) | 1091 (23.0) |  |
| **Men** | 7359 (65.7) | 4308 (67.0) | 3051 (63.8) | 0.066 | 4247 (65.8) | 3133 (66.0) | 0.004 |
| **CHA_2_DS_2_-VASc** | 3.3±1.7 | 3.2±1.7 | 3.56±1.5 | 0.238 | 3.4±1.7 | 3.4±1.6 | 0.013 |
| **CHA_2_DS_2_-VASc ≥3** | 7559 (67.4) | 3992 (62.1) | 3567 (74.6) |  | 4376 (67.8) | 3243 (68.4) |  |
| **Charlson Comorbidity Index** | 3.5±2.2 | 3.5±2.2 | 3.5±2.2 | 0.039 | 3.5±2.2 | 3.5±2.2 | 0.007 |
| **Charlson Comorbidity Index ≥3** | 7024 (62.7) | 3974 (61.8) | 3050 (63.8) |  | 4088 (63.3) | 2967 (62.5) |  |
| **Hypertension** | 9266 (82.7) | 5112 (79.5) | 4154 (86.9) | 0.198 |  |  | 0.018 |
| **Diabetes** | 2643 (23.6) | 1423 (22.1) | 1220 (25.5) | 0.079 | 1543 (23.9) | 1146 (24.2) | 0.005 |
| **Dyslipidemia** | 5966 (53.2) | 3217 (50.0) | 2749 (57.5) | 0.150 | 3459 (53.6) | 2590 (54.6) | 0.019 |
| **Heart failure** | 4405 (39.3) | 2441 (38.0) | 1964 (41.1) | 0.063 | 2535 (39.3) | 1863 (39.3) | <0.001 |
| **Prior stroke** | 2246 (20.0) | 1330 (20.7) | 916 (19.2) | 0.038 | 1300 (20.1) | 971 (20.5) | 0.008 |
| **Prior myocardial infarction** | 568 (5.1) | 332 (5.2) | 236 (4.9) | 0.010 | 325 (5.0) | 239 (5.0) | <0.001 |
| **Peripheral artery disease** | 2588 (23.1) | 1378 (21.4) | 1210 (25.3) | 0.091 | 1493 (23.1) | 1103 (23.3) | 0.002 |
| **Chronic kidney disease** | 411 (3.7) | 275 (4.3) | 136 (2.9) | 0.077 | 239 (3.7) | 184 (3.9) | 0.009 |
| **COPD** | 543 (4.8) | 363(5.7) | 180 (3.8) | 0.088 | 310 (4.8) | 224 (4.7) | 0.003 |
| **Cancer** | 593 (5.3) | 328(5.1) | 265 (5.5) | 0.019 | 339 (5.3) | 246 (5.2) | 0.002 |
| **Antiplatelet use** |  |  |  | 0.278 |  |  | 0.006 |
| **None** | 7722 (68.9) | 3983 (61.9) | 3739 (78.2) |  | 4448 (68.9) | 3233 (68.1) |  |
| **Aspirin only** | 1898 (16.3) | 1367 (21.3) | 531 (11.1) |  | 1087 (16.9) | 812 (17) |  |
| **P2Y12 only** | 616 (5.5) | 394 (6.1) | 222 (4.6) |  | 355 (5.5) | 264 (5.6) |  |
| **Both** | 974 (8.7) | 686 (10.7) | 288 (6.0) |  | 564 (8.7) | 437 (9.2) |  |
| **Body weight, kg** | 66.5±11.5 | 66.4±11.5 | 66.5±11.5 | 0.008 | 66.4±11.5 | 66.8±11.7 | 0.030 |
| **Body weight ≤60 kg** | 8049 (71.8) | 4601 (71.6) | 3448 (72.1) |  | 4616 (71.5) | 3441 (72.5) |  |
| **BMI kg/m^2^** | 24.9±3.3 | 24.8±3.3 | 25.1±3.3 | 0.095 | 25.0±3.54 | 24.97±3.3 | 0.003 |
| **eGFR, ml/min/1.73m^2^** | 81.6±44.3 | 81.6±42.6 | 81.5±46.5 | 0.003 | 81.6±46.8 | 81.6±41.6 | <0.001 |
| **eGFR ≤60 ml/min/1.73m^2^** | 1649 (14.7) | 961 (15.0) | 688 (14.4) |  | 1004 (15.6) | 657 (13.8) |  |
| **eGFR category** |  |  |  |  |  |  |  |
| **15-29 ml/min/1.73m^2^** | 58 (0.5) | 46 (0.7) | 12 (0.3) |  | 41 (0.6) | 14 (0.4) |  |
| **30-44 ml/min/1.73m^2^** | 231 (2.1) | 157 (2.4) | 74 (1.6) |  | 154 (2.4) | 69 (1.4) |  |
| **45-59 ml/min/1.73m^2^** | 1360 (12.1) | 758 (11.8) | 602 (12.6) |  | 809 (12.5) | 574 (12.1) |  |
| **60-89 ml/min/1.73m^2^** | 6735 (60.1) | 3764 (58.5) | 2971 (62.2) |  | 3822 (59.2) | 2893 (61.0) |  |
| **≥90 ml/min/1.73m^2^** | 2826 (25.2) | 1705 (26.5) | 1121 (23.5) |  | 1628 (25.2) | 1195 (25.2) |  |
| **Heavy drinkers*** | 4391 (39.2) | 2632 (40.9) | 1759 (36.8) | 0.084 | 2528 (39.2) | 1852 (39.0) | 0.002 |
| **Current smokers** | 1696 (15.1) | 1161 (18.1) | 535 (11.2) | 0.195 | 974 (15.1) | 726 (15.3) | 0.005 |
| **Regular exercise** | 2576 (23.0) | 1483 (23.1) | 1093 (22.9) | 0.004 | 1490 (23.1) | 1089 (23.0) | 0.003 |
| **Rivaroxaban dose** |  |  |  |  |  |  |  |
| **20mg once daily** |  | N/A | 2153 (45.0) |  | N/A | 2092 (44.1) |  |
| **15mg once daily** |  | N/A | 2627 (55.0) |  | N/A | 2653 (55.9) |  |

Continuous variables are shown as mean and standard deviation. Categorical variables are presented as number (percentage).

*Heavy drinker was defined as alcohol consumption ≥30g/day.

Abbreviation: ASD, absolute standardized difference; BMI, body mass index; COPD, chronic obstructive pulmonary disease; eGFR, estimated glomerular filtration rate; IPTW, inverse probability of treatment weighting; N/A, not available.

**Supplementary Figure**

**Supplementary Figure 1. Distribution of propensity scores in warfarin and rivaroxaban groups after IPTW (,main analysis set)**


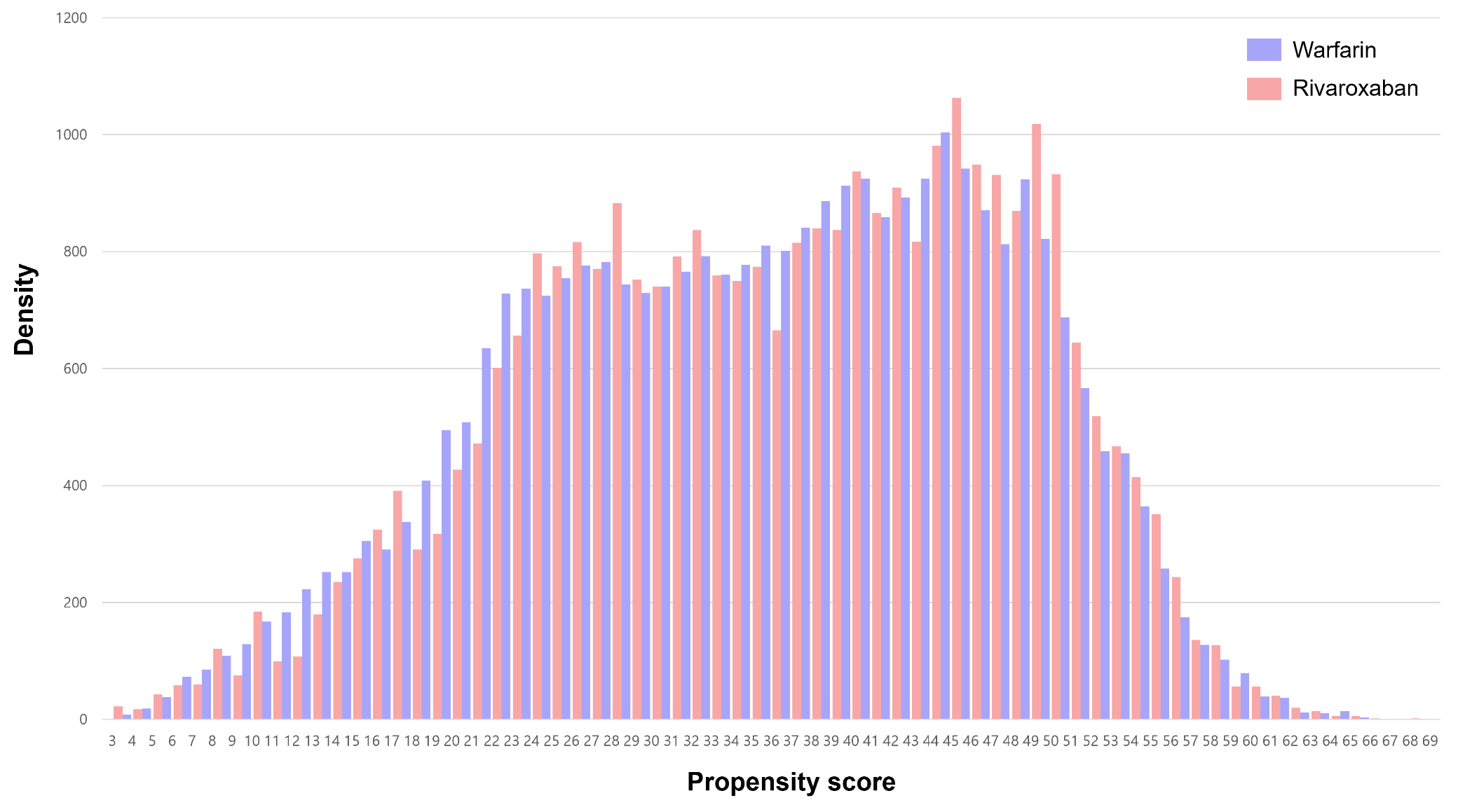


**Supplementary Figure 2. Various sensitivity analyses for the primary outcome (kidney failure)**

**
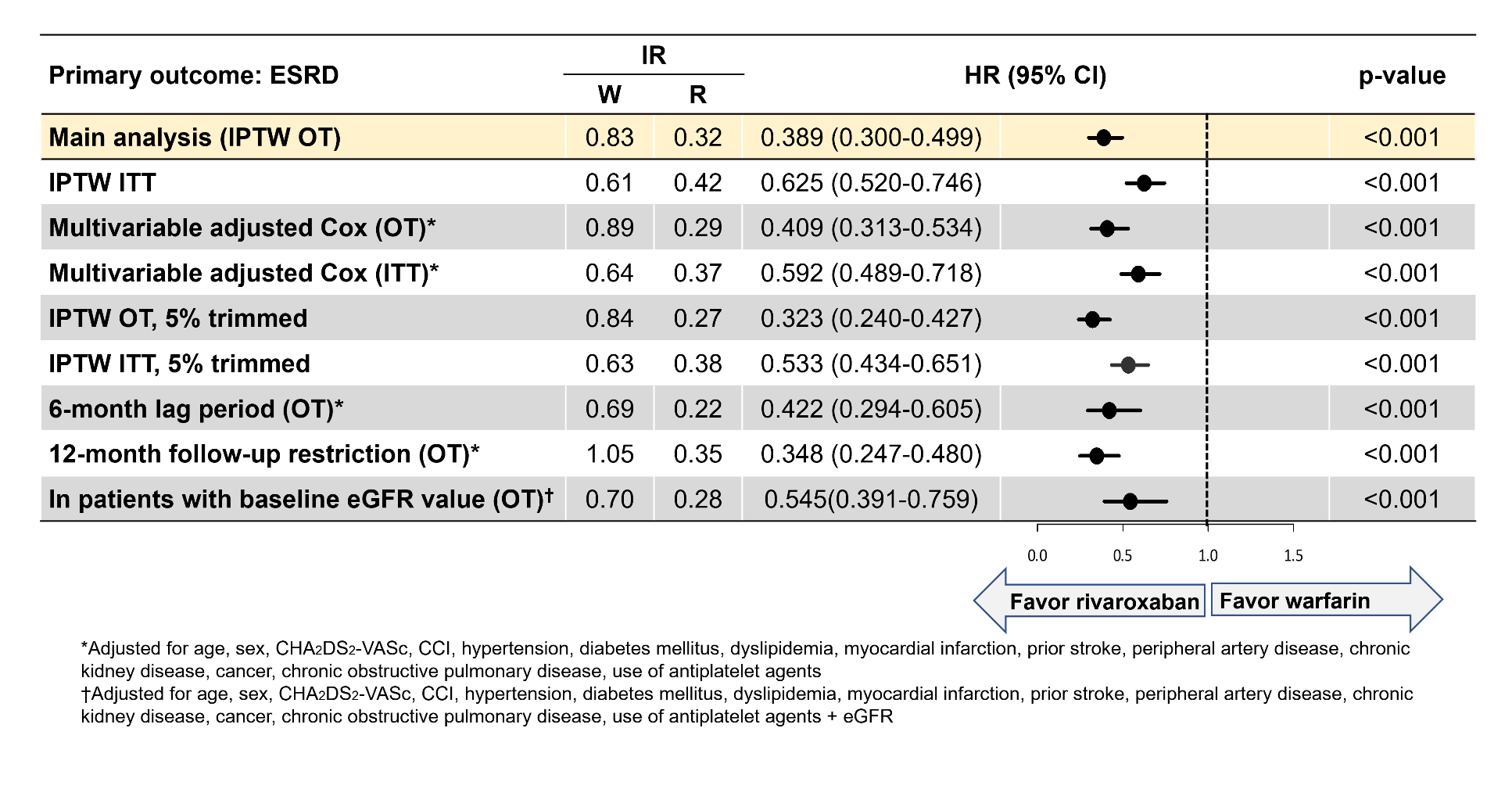
**

Incidence rate, per 100 person-years

Abbreviations: CI, confidence interval; eGFR, estimated glomerular filtration rate; HR, hazard ratio; IPTW, inverse probability of treatment weighting; ITT, intention-to-treat; OT, on-treatment; R, rivaroxaban; W, warfarin.
